# Biological Characterization, Domestication, and the Molecular Basis of Iron Enrichment in a Wild *Flammulina yunnanensis* Strain

**DOI:** 10.3390/jof12080551

**Published:** 2026-07-23

**Authors:** Yuanchao Liu, Xinyu Shi, Yifan Li, Chenwei Wu, Manjun Cai, Xiaoxian Wu, Tamdrin Tsering, Yutao Wu, Ruihan Wang, Ming Jiang, Huiping Hu

**Affiliations:** 1 State Key Laboratory of Applied Microbiology Southern China, National Health Commission Science and Technology Innovation Platform for Nutrition and Safety of Microbial Food, Guangdong Provincial Key Laboratory of Microbial Safety and Health, Institute of Microbiology, Guangdong Academy of Sciences, Guangzhou 510070, China; liuyc1020@163.com (Y.L.);; 2 Key Laboratory of Big Data Technologies for Food Microbiological Safety, State Administration for Market Regulation, NHC Specialty Laboratory of Food Safety Risk Assessment and Standard Development, Guangdong-Hong Kong-Macao Greater Bay Area Microbiological Safety and Health International Science and Technology Innovation Center, Guangzhou 510070, China; 3 College of Life Science and Technology, Mudanjiang Normal University, Mudanjiang 157012, China; 4 Guangdong Yuewei Biotechnology Co., Ltd., Shaoguan 512029, China

**Keywords:** *Flammulina yunnanensis*, domestication, nutritional composition, iron metabolism, FTR1 iron permease, comparative genomics

## Abstract

In this paper, we reported a wild strain Z68, isolated from Tibet, was identified as *Flammulina yunnanensis* based on ITS sequence and phylogenetic analysis. Optimal condition for its mycelial growth (maltose, yeast extract, magnesium sulfate, 20 °C and pH 7.0–8.0) and fruiting ability was confirmed. Its fruiting bodies contained 25.1 g/100 g protein and 19.80 g/100 g of 16 amino acids, with iron, potassium and zinc, contents significantly higher than these in the controls *F. fennae* and *F. velutipes*. Significantly, whole-genome sequencing and comparative analysis of iron-metabolism gene families were performed between *F. yunnanensis*, *F. fennae* and *F. velutipes* to elucidate the basis of its notably elevated iron content, revealing most single copied and conserved iron-related gene families and two-fold expanded iron permease FTR1 in *F. yunnanensi*, which is the iron–sulfur cluster assembly protein family. Sequence, topology (DeepTMHMM), and structural-confidence (ColabFold/AlphaFold2) analyses of FTR1 identified six species-specified physicochemical substitutions, of which a charge reverse at the intracellular channel (Glu→Lys) and a polar-to-nonpolar change in the TM3 pore (Ser→Ala) were predicted to enhance iron transport efficiency. These suggested a possible two-tier mechanism underlying the elevated iron content of *F. yunnanensis*, combining gene-family expansion and predicted functional fine-tuning of the FTR1 permease. This study provided insight for biological characterization, domestication, and the molecular basis of iron enrichment in a *F. yunnanensis* Z68.

## 1. Introduction

The genus Flammulina comprises a group of edible macrofungi, such as *F. yunnanensis*, *F. velutipes*, and *F. velutipes*. In which, *F. velutipes* is one of the most important cultivated edible mushrooms in China and Japan. According to the color of the fruiting body, the cultivars available on the market are mainly divided into two major groups, including white and yellow. *F. velutipes* is characterized by its crisp texture and rich nutrients [1,2], and it occupies a dominant position in the early markets, among which the strain “Zajiao 19”, bred by the Sanming Institute of Mycology (Sanming, Fujian, China), once held the largest market share [3].

Subsequently, white *F. velutipes* introduced from Japan rapidly became the main cultivars in industrialized production due to their shorter production cycle and higher yield [4,5,6]. Currently, some commercial white enoki strains in China still rely on imports [7]. Researchers have successfully bred a series of high-yield cultivars, such as Shangyan A130, F1P8, and Chuanjin 33, through breeding techniques including selfing, hybridization, and mutagenesis and provided important support for the large-scale production of *F. velutipes* [6,8,9,10,11].

*Flammulina* are rich in proteins, amino acids, sesquiterpenes, polysaccharides, flavonoids, and other nutritional components, and possess various pharmacological activities [12,13], including anticancer, antibacterial, antioxidant, and immunoregulatory effects [14,15,16]. Meanwhile, iron was an important nutrient for human, which was provided through food intaking. Given that edible fungi are significant and future food, their iron content and metabolism was focused, especially for *F. yunnanensis*. However, the species of cultivating *Flammulina* were rare. Thus, exploring wild Flammulina germplasm is needed.

China is exceptionally rich in wild edible mushroom resources, with over 9300 macro-fungal species identified nationwide [17]. Among all provinces, Yunnan stands out as the undisputed leader: it hosts 2753 known wild fungal species across 124 families, 599 genera, accounting for 57.4% of the country’s total known macro-fungi [18], and has contributed 314 new species—35% of all new species described from China [19]. Most remarkably, Yunnan boasts 900 wild edible mushroom species, representing 36% of the world’s edible fungi and 90% of China’s edible fungal diversity, firmly establishing its global significance as a hotspot for wild edible mushroom resources [18]. These findings identify southwestern China as an important reservoir of wild edible mushroom germplasm, including potentially diverse and underexplored wild *Flammulina* resources [19,20].

Within the genus *Flammulina*, this feature is likewise particularly evident. According to the Index Fungorum database (accessed in March 2026), the type materials of taxa such as *F. yunnanensis*, *F. velutipes*, and *F. velutipes* [21] were all collected from Yunnan, China. This indicates that southwestern China is not only a hotspot for discovering new edible mushroom species but also harbors abundant wild germplasm resources of the genus *Flammulina*, which remain to be further collected, evaluated, and exploited.

To further expand our edible mushroom germplasm resources, especially *Flammulina* genus, this study focused on a wild strain of *F. yunnanensis* isolated from Tibet. By optimizing solid-medium culture conditions, the optimal conditions for mycelial growth were identified. Preliminary fruiting cultivation was carried out to determine its fruiting ability. The nutritional contents of the fruiting bodies were analyzed to examine its nutrient value and commercial capability, in which iron was focused. Whole-genome sequencing and comparative analysis were performed with *F. fennae* and *F. velutipes* as controls to elucidate the basis of its notably nutrients since they are the dominant species for cultivation. Topology (DeepTMHMM), and structural-confidence (ColabFold/AlphaFold2) analyses were carried out to explore the mechanism of its abundant nutrient. This work may provide a new material and a basis for the collection, preservation, and utilization of *Flammulina* germplasm resources.

## 2. Materials and Methods

### 2.1. Strains

The Z68 strain, was obtained by tissue isolation and purification from a wild fruiting body of *F. yunnanensis* collected in Tibet and then preserved at the Edible Fungi Research and Development Center, Institute of Microbiology, Guangdong Academy of Sciences (Guangzhou, China). The control strains, *F. fennae* M58 and *F. velutipes* FV0027, were also preserved at the same center.

### 2.2. Main Reagents

The main reagents used in this study included potassium dihydrogen phosphate, magnesium sulfate, calcium chloride, calcium carbonate, and ferrous sulfate; tryptone, yeast extract powder, beef extract, urea, and acid-hydrolyzed casein peptone; as well as glucose, fructose, sucrose, lactose, and maltose. All reagents were of analytical grade and purchased from Huankai Microbial Com. (Guangzhou, China).

### 2.3. Basic Medium

The basal medium was prepared following the formulation reported by He et al. [20] with slight modifications, consisting of glucose 20 g/L, tryptone 2 g/L, potassium dihydrogen phosphate 0.5 g/L, agar powder 16 g/L, and distilled water to 1000 mL, with natural pH retained. The medium was sterilized at 121 °C for 20 min before use.

### 2.4. Determination of Optimal Culture Conditions for Mycelial Growth

The methods reported by Zhang et al. [22] were followed with modifications to investigate the effects of five factors, namely carbon source, nitrogen source, inorganic salt, temperature, and pH, on mycelial growth. Vigorous Z68 mycelial plugs (5 mm × 5 mm) were inoculated in the center of each treatment medium. Except for the temperature experiment, all cultures were incubated in the dark at 20 °C. Each treatment was performed with five replicates. After inoculation, colony diameter was measured every 3 days using the cross-measurement method until the mycelia in one treatment had fully covered the Petri dish. Mycelial growth rate (mm/d) was calculated as colony diameter (mm) divided by cultivation time (d) [23]. Meanwhile, mycelial vigor and density were observed and recorded. Mycelial density was scored at—to ++++ levels (no-growth, −; thin, +; moderate, ++; dense, +++; compact, ++++).

#### 2.4.1. Carbon Source Test

In the carbon source experiment, glucose, maltose, fructose, sucrose, and lactose were used to replace the carbon source in the basal medium at an equal carbon concentration (20 g/L), while a medium without any added carbon source was used as the control.

#### 2.4.2. Nitrogen Source Test

In the nitrogen source experiment, tryptone, yeast extract, beef extract, urea, and acid-hydrolyzed casein were used to replace the nitrogen source in the basal medium at an equal nitrogen concentration (2 g/L), while a medium without any added nitrogen source was used as the control.

#### 2.4.3. Inorganic Salt Test

In the inorganic salt experiment, potassium dihydrogen phosphate, magnesium sulfate, calcium chloride, calcium carbonate, and ferrous sulfate were individually added to the basal medium lacking the corresponding inorganic salt, each at a concentration of 0.5 g/L, while a medium without any added inorganic salt served as the control.

#### 2.4.4. pH Test

In the pH experiment, the pH of the basal medium was adjusted to 4.0, 5.0, 6.0, 7.0, 8.0, 9.0, and 10.0 using 1 mol/L HCl or NaOH, while medium at its natural pH (approximately 6.5) was used as the control.

#### 2.4.5. Temperature Testing

In the temperature experiment, inoculated plates were incubated at 12 °C, 16 °C, 18 °C, 20 °C, 22 °C, 25 °C, and 28 °C, with the conventional cultivation temperature of 25 °C used as the control.

### 2.5. Molecular Phylogenetic Analysis

Generally, amplification and direct sequencing of fungal ribosomal RNA genes for phylogenetics was carried out with ITS1 TCCGTAGGTGAACCTGCGG and ITS4 TCCTCCGCTTATTGATATGC as primers with a standard PCR protocol [24]. ITS sequences of *Flammulina* species and related outgroup taxa were downloaded from the NCBI database. Phylogenetic analysis was performed using MEGA software (Megasoftware, v12.1.2), and a phylogenetic tree was constructed using the maximum likelihood (ML) method. The optimal substitution model was determined as K2+G using the built-in “Find Best DNA Model” function. Branch support was evaluated by 1000 bootstrap replicates under the ML method. *Cyptotrama asprata* was selected as the outgroup for tree construction [25].

### 2.6. Domestication and Cultivation

The cultivation substrate consisted of 50% cottonseed hulls, 33% mixed wood sawdust, 15% wheat bran, 1% calcium carbonate, and 1% sucrose (by weight). The cultivation substrate was packed into cultivation bags and sterilization was achieved by maintaining a pressure of 0.15 MPa for 2 h. and then cooled to room temperature before use. Under aseptic conditions, the tested strain was inoculated onto the surface of the cultivation substrate. The bags were incubated in the dark at 20 °C for spawn running. After the mycelia had fully colonized the bags, the inoculum block on the surface was removed, and the bags were transferred to 15 °C for primordium induction. Once primordia appeared, the temperature was further reduced to 10 °C and the air humidity was maintained at 90%. Fruiting bodies were harvested at maturity and used for nutritional analysis.

### 2.7. Nutritional Analysis and Testing

To scientifically evaluate the nutritional quality of the wild *F. yunnanensis* strain Z68, this strain was designated as the test strain in the present study. *F. fennae* M58 and *F. velutipes* FV0027 were selected as control strains. These two control strains were chosen because, on the one hand, both are taxonomically well-defined and representative cultivated *Flammulina* strains whose nutritional data provide a reliable basis for comparison; on the other hand, both are common commercial types on the market and represent different cultivation categories [22,26]. Comparison with these strains allows for an objective evaluation of the characteristics of Z68 in terms of nutritional components such as protein, amino acids, and mineral elements, and further reveals its potential application value. The tests were based on dried weight and mass.

#### 2.7.1. Determination of Major Nutrients and Amino Acid Content in Fruiting Bodies

After drying, the fruiting bodies of *Flammulina* were uniformly pulverized. Protein content was determined using an automatic Kjeldahl analyzer (Hanon, Jinan, China) following Method I (Kjeldahl method) of the national food safety standard GB 5009.5-2025 [27] (“Determination of protein in foods”). Fat extraction and determination were performed using a Soxhlet extractor (Huankai Biotechnology, Guangzhou, China) following Method I (Soxhlet extraction method) of GB 5009.6-2025 [28] (“Determination of fat in foods”). Dietary fiber content was determined using high-performance liquid chromatography (HPLC, Shimadzu, Kyoto, Japan) following the enzymatic hydrolysis method specified in GB 5009.88-2023 [29] (“Determination of dietary fiber in foods”). Amino acid content in the samples was determined using an amino acid analyzer (Hitachi, Tokyo, Japan. ion-exchange chromatograph with post-column ninhydrin derivatization) as specified in GB 5009.124-2016 [30] (“Determination of amino acids in foods”).

#### 2.7.2. Determination of Mineral Element and Vitamin Content in Fruiting Bodies

The mineral element contents of the fruiting bodies were determined in accordance with GB5009.268-2025 [31], National Food Safety Standard—Determination of Multiple Elements in Foods, using Method I, inductively coupled plasma mass spectrometry (ICP-MS, Thermo Fisher, Germany). Vitamin contents were determined in accordance with GB5009.84-2016 [32], National Food Safety Standard—Determination of Vitamin B1 in Foods, using Method I, high-performance liquid chromatography (HPLC, Shimadzu, Japan); GB5009.85-2016 [33], National Food Safety Standard—Determination of Vitamin B2 in Foods, using Method I, HPLC; and GB5009.86-2016 [34], National Food Safety Standard—Determination of Ascorbic Acid in Foods, using Method I, HPLC.

### 2.8. DNA Extraction, Sequencing and Assembly

#### 2.8.1. Culture Conditions for Z68 Mycelium

The *F. yunnanensis* Z68 strain was cultured on Potato Dextrose Agar (PDA) medium. The PDA medium was prepared by dissolving 200 g of potato, 20 g of glucose, and 20 g of agar per liter of distilled water. The cultures were incubated at 20 °C until the mycelium had fully colonized the surface of the Petri dish (purchased from Huankai Microbial Com. Guangzhou, China). Once the entire surface was covered, the mycelium was transferred to fresh PDA plates and incubated for 8 days. The mycelium was then collected into 2 mL grinding tubes, immediately frozen in liquid nitrogen, and stored at −80 °C for subsequent experiments.

#### 2.8.2. DNA Extraction and Genome Sequencing

Following a standardized protocol, genomic DNA from Z68 (*F. yunnanensis*) and M58 (*F. fennae*) were extracted using Fungal DNA Extraction Mini Kit B (Guangzhou Mabio Biotechnology Co., Ltd., Guangzhou, China) [35]. The quality and purity of the extracted DNA were assessed using 0.5% agarose gel electrophoresis and the NanoDrop 2000 spectrophotometer (Thermo Scientific, Wilmington, DE, USA). High-quality whole-genome sequencing was obtained by combining Illumina and PacBio sequencing technologies. A short-read library (about 300 bp) was constructed on the Illumina NovaSeq (Illumina Inc., San Diego, CA, USA) platform, and a long-read library (about 20 kb) was constructed on the PacBio HiFi (CCS) platform (Pacific Biosciences, Menlo Park, CA, USA). Raw Illumina paired-end reads were quality controlled and filtered using fastp v0.22.0 with the following parameters: -q 15 -u 40 -y -g -Y 10 --adapter_fasta adapter.fa -l 100 -b 150 -B 150. Adapter sequences specified in adapter.fa were removed, and 3′ polyG-tail trimming was enabled using -g. Reads were discarded when more than 40% of their bases had Phred quality scores below 15 (-q 15 -u 40). Low-complexity filtering was enabled, with the sequence-complexity threshold set to 10% (-y -Y 10). Reads shorter than 100 bp after processing were discarded (-l 100). The maximum lengths of both R1 and R2 reads were restricted to 150 bp, and sequences extending beyond this limit were truncated from the 3′ end (-b 150 -B 150). The resulting high-quality paired-end reads were used for subsequent genome assembly. PacBio HiFi long reads provided in BAM format were demultiplexed according to sample-specific barcode sequences using PacBio lima v2.12.0, the HiFi preset appropriate for the barcode design: lima --same --split-bam-named. Basic sequencing statistics, including the total number of reads, total number of bases, mean read length, and read-length N50, were calculated for each demultiplexed sample. Because the delivered reads were already CCS/HiFi reads, no additional quality filtering, length filtering, or sequence trimming was performed after demultiplexing, and the reads were used directly for genome assembly.

#### 2.8.3. Genome Assembly and Annotation

The PacBio CCS reads of Z68 (*F. yunnanensis*, abbreviated as Flaspp) and M58 (*F. fennae*, control strain, abbreviated as Flafe) were assembled using Hifiasm (v0.19.9) [36] with default parameters to obtain the genome assembly of strain Z68. Subsequently, Illumina short reads were used for error correction with Pilon [37], yielding a final contig-level genome assembly of *F. yunnanensis*. The completeness of the assembled genome was assessed using BUSCO (v5.1.2) [38] against the fungal lineage dataset fungi_odb10 in consideration of its generality. The genome of *F. velutipes* (control strain, abbreviated as Flave) was downloaded from NCBI (accession GCA_011800155.1, reference genome). RepeatModeler (v2.0.5) [39] and RepeatMasker (v1.0) [40] were used to identify and mask repetitive sequences in the genome. First, de novo structure prediction was performed using RepeatModeler (v 2.0.1) to construct a species-specific repeat library. Then, RepeatMasker was used to mask repetitive regions based on this library, and the RepBase database was employed to identify known repeat sequences. Subsequently, de novo gene prediction was performed using the Helixer (v0.3.4) [41] online server (https://www.plabipd.de/helixer_main.html (accessed on 2 April 2026)). In parallel, Annevo (v2.2.2) [42] software was used for auxiliary gene prediction. The results from multiple prediction sources were integrated using EVidenceModeler (EVM, v2.1.0), and protein sequences were extracted using gffread (v0.12.7). Functional annotation was performed using the funannotate software (1.8.17) [43], which integrates multiple toolkits and database alignment strategies. This included orthologous group (OG) functional classification using EggNOG-mapper (v2.1.13) [44] combined with DIAMOND (v2.1.24.178) [45]; carbohydrate-active enzyme annotation based on the dbCAN (v5.2.8) database to identify CAZymes families [46]; sequence similarity searches of predicted gene models against public databases including UniProt/Swiss-Prot (v 2026_02) [47], and the KEGG pathway database [48]; and protein domain annotation using Pfam (v 38.1) [49] and InterProScan (v 5.77-108.0) [50] to identify conserved domains and functional sites. Secondary metabolite gene clusters were predicted using the antiSMASH online tool (v 8.0) [51].

#### 2.8.4. Iron Metabolism Gene Family Classification and Copy Number Analysis

Iron-related gene families were identified using eggNOG-mapper v2 (Diamond mode, E-value ≤ 1 × 10^−5^, eggNOG 5.0 database; GO:0006879, GO:0055072, GO:0016226), antiSMASH v8.0.4 (NI-siderophore BGC focus), and keyword searches on CDS annotations. Orthologous clustering was performed with OrthoFinder v2.5 (Diamond; MCL I = 1.5). Because fewer than four taxa were available (CAFE5 minimum), copy number differences were quantified by direct counting and fold-change.

#### 2.8.5. FTR1 Iron Permease Sequence and Structural Analysis

FTR1 protein sequences from all three species were aligned with MAFFT v7 (L-INS-i, BLOSUM62, gap-open 1.53); residues unique to *F. yunnanensis* relative to both controls were classified by physicochemical property change. Because *F. yunnanensis* carries a one-residue indel in the N-terminal loop (positions 40–75), alignment coordinates ≥ 41 are offset by −1 relative to the *F. yunnanensis* raw sequence index. Each variable position was assigned a topological context (TMhelix/inside/outside) using DeepTMHMM (v1.0) [52], providing the structural framework for interpreting functional consequences. Per-residue structural confidence (pLDDT) was obtained from ColabFold v1.5.5 (MMseqs2 + AlphaFold2-ptm, num_recycles = 3, five models per sequence; top-ranked model selected by pTM score) [53,54]; pLDDT values are stored in the B-factor column of each output PDB file.

### 2.9. Statistical Analysis

Statistical analysis and graphing were performed using Excel 2019.

## 3. Results

### 3.1. Phylogenetic Tree

**Figure 1:**
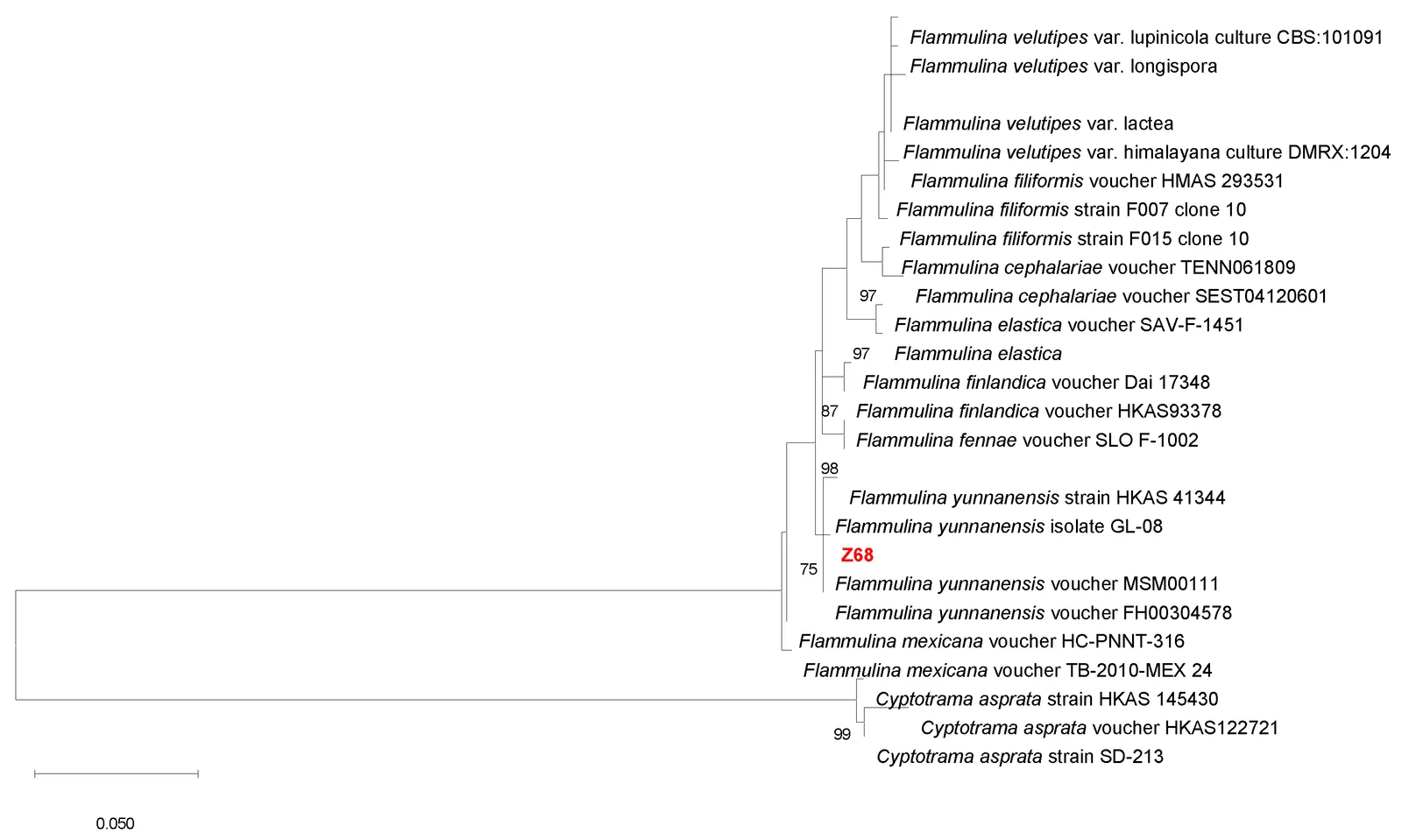
Phylogenetic tree of the genus *Flammulina* based on maximum likelihood (ML) analysis of ITS sequence. ML bootstrap only shows branches with support values greater than 50%.

The maximum likelihood phylogenetic tree constructed based on ITS sequences showed that strain Z68 clustered within the same branch as the reference strains of *F. yunnanensis* (Figure 1). This branch was supported by a relatively high bootstrap value (75%) and was clearly separated from other species within the genus *Flammulina*. Accordingly, strain Z68 was preliminarily identified as *F. yunnanensis* Z.W. Ge & Zhu L. Yang.

### 3.2. Study of Biological Properties

#### 3.2.1. Colony Growth Phenotype

Biological characteristics of the strain were further investigated through a series of single-factor experiments to evaluate the effects of different culture conditions on mycelial growth.

**Figure 2:**
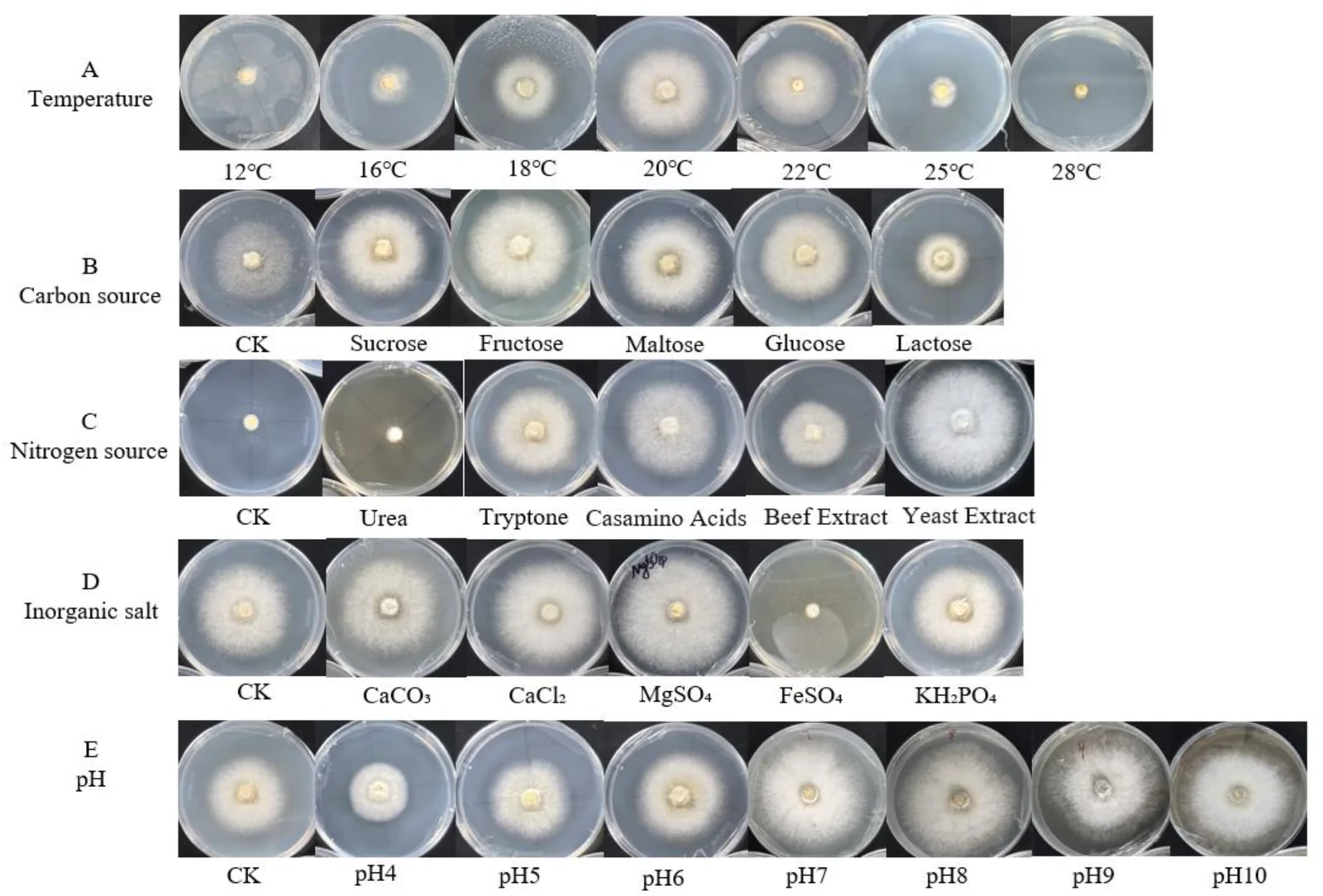
Colony morphology of strain Z68 under various single-factor culture conditions. CK means basal medium. (**A**) Temperature test; (**B**) Carbon source test; (**C**) Nitrogen source test; (**D**) Inorganic salt test; (**E**) pH test.

The temperature experiment showed that Z68 mycelia grown at 20 °C were dense and vigorous (Figure 2A), whereas mycelial growth was completely inhibited at 28 °C. In the absence of an added carbon source, the mycelia of Z68 grew sparsely. Under different carbon source conditions, Z68 grew vigorously and formed dense mycelia on media containing maltose, glucose, or fructose, whereas mycelia on lactose medium were prone to aging (Figure 2B), indicating that carbon source is an important factor affecting the mycelial growth of Z68. In the absence of an added nitrogen source, Z68 mycelia were also very sparse. Among different nitrogen sources, mycelia grew most vigorously and densely on medium containing yeast extract, whereas growth was poorest on medium containing urea (Figure 2C), indicating that nitrogen source is another important factor influencing mycelial growth and that the strain preferentially utilizes organic nitrogen sources. Among the different inorganic salt treatments, magnesium sulfate was the most favorable for rapid mycelial growth, whereas ferrous sulfate showed a clear inhibitory effect (Figure 2D). The pH experiment showed that Z68 mycelia were able to grow over a wide pH range of 4.0–10.0. Growth was better under alkaline than acidic conditions, with the best performance observed at neutral to slightly alkaline pH values of 7.0~8.0 (Figure 2E).

#### 3.2.2. Effect of Carbon Sources on the Mycelial Growth Rate of the *F. yunnanensis* Strain Z68

As shown in Table 1, carbon source significantly affected both the radial growth and hyphal density of Z68. Maltose produced the highest growth rate (7.52 ± 0.50 mm/d), which was significantly higher than those obtained with glucose and the basal medium at *p* < 0.05, but did not differ significantly from fructose or sucrose. However, the mycelia grown on maltose were dense (+++) rather than compact (++++). Fructose produced a similarly high growth rate (7.27 ± 0.31 mm/d) and the highest hyphal density (++++), indicating a more favorable balance between rapid radial extension and biomass accumulation. Glucose also produced compact mycelia (++++) despite a significantly lower growth rate than maltose. In contrast, lactose resulted in the lowest growth rate (4.11 ± 0.25 mm/d), significantly lower than all other treatments at *p* < 0.01, although the colony remained relatively dense (+++). The basal medium supported moderate radial growth but only sparse mycelia (+). These results indicate that growth rate and hyphal density responded differently to carbon sources and suggest that fructose was more suitable for overall vegetative growth, whereas maltose primarily promoted radial extension.

#### 3.2.3. The Effect of Nitrogen Sources on the Mycelial Growth Rate of the *F. yunnanensis* Strain Z68

As shown in Table 2, nitrogen source markedly influenced both mycelial growth rate and colony morphology (Table 2). Yeast powder produced the highest growth rate (8.82 ± 0.20 mm/d), which was significantly higher than all other treatments at *p* < 0.01, and simultaneously resulted in compact mycelia (++++). Tryptone ranked second in growth rate (6.93 ± 0.25 mm/d) and produced dense colonies (+++), indicating that it was also readily utilized by Z68. Acid-hydrolyzed casein and beef extract supported intermediate growth rates of 6.45 ± 0.33 and 5.99 ± 0.46 mm/d, respectively, but generated only moderately dense mycelia (++). In contrast, urea and the nitrogen-free control showed the lowest growth rates, with no significant difference between them, and both produced sparse colonies (+). The poor performance of urea suggests that it was not efficiently assimilated by Z68 under the tested conditions. Overall, yeast powder was the most suitable nitrogen source because it promoted both rapid radial growth and compact mycelial development, probably owing to its readily available amino acids, peptides, vitamins, and other growth factors.

#### 3.2.4. The Effect of Inorganic Salts on the Mycelial Growth Rate of *F. yunnanensis* Strain Z68

As shown in Table 3, Inorganic salts affected radial growth and hyphal density differently. Calcium chloride produced the highest growth rate (8.34 ± 0.22 mm/d), followed closely by magnesium sulfate (8.23 ± 0.50 mm/d), with no significant difference between these two treatments. Nevertheless, magnesium sulfate produced compact mycelia (++++), whereas calcium chloride produced only dense mycelia (+++). Thus, although calcium chloride slightly favored radial extension, magnesium sulfate was more effective in promoting overall mycelial development and colony compactness. The basal medium, potassium dihydrogen phosphate, and calcium carbonate supported similar growth rates of 7.21–7.53 mm/d and all produced dense colonies (+++), indicating that these salts had limited stimulatory effects under the tested conditions. By contrast, ferrous sulfate reduced the growth rate to 0.74 ± 0.94 mm/d, significantly lower than all other treatments at *p* < 0.01, and completely inhibited visible mycelial development (−). This strong inhibition may be associated with Fe^2+^ toxicity and iron-induced oxidative stress. Therefore, magnesium sulfate appeared to be the most favorable inorganic salt when both radial growth and hyphal density were considered, whereas ferrous sulfate was strongly inhibitory at the tested concentration.

#### 3.2.5. Effect of pH on the Mycelial Growth Rate of the Z68 Strain of *F. yunnanensis*

As shown in Table 4, the pH of the medium significantly affected both radial growth and hyphal density of Z68. The mycelial growth rate generally increased as the pH rose from 4.5 to 8.0, decreased slightly at pH 9.0, and then reached its maximum at pH 10.0. The lowest growth rate was observed at pH 4.5 (4.52 ± 0.23 mm/d), whereas the highest was recorded at pH 10.0 (8.03 ± 0.24 mm/d), with a highly significant difference between these treatments (*p* < 0.01). Growth at pH 7.0–10.0 was generally faster than that under acidic conditions, indicating that Z68 tolerated neutral-to-alkaline environments better than strongly acidic conditions. However, radial growth rate and hyphal density did not respond identically to pH. The natural-pH control produced the most compact mycelia (++++), despite its intermediate growth rate. At pH 7.0 and 8.0, the colonies combined relatively rapid growth with dense mycelia (+++), whereas pH 9.0 and 10.0 supported rapid but only moderately dense growth (++). These findings suggest that pH 7.0–8.0 was more suitable for balanced vegetative growth, while pH 10.0 primarily promoted radial extension rather than compact mycelial development.

#### 3.2.6. The Effect of Temperature on the Mycelial Growth Rate of the Z68 Strain of *F. yunnanensis*

As shown in Table 5, temperature significantly affected both the growth rate and hyphal density of Z68. The optimal temperature for mycelial growth was 20 °C, where the growth rate peaked at 6.79 ± 0.12 mm/d (marked with ‘A’, *p* < 0.01) and hyphal density reached the maximum (++++). Growth rates declined sharply at higher temperatures: at 25 °C, the rate dropped to 2.75 ± 0.09 mm/d (‘F’, *p* < 0.01), and no growth was observed at 28 °C (0 mm/d, ‘G’). Notably, although 18 °C and 22 °C also supported relatively high growth rates (5.44 and 6.15 mm/d, respectively), they were significantly lower than at 20 °C. Hyphal density followed a similar trend—dense growth (+++) occurred at 16–22 °C, while sparse (+), moderate (+++), or compact (++++) densities correlated with increasing growth rates. These results indicate that Z68 exhibits a narrow thermal optimum centered at 20 °C, suggesting it may be adapted to cooler environments and is sensitive to heat stress above 25 °C.

### 3.3. Domestication and Cultivation: Management of Fruiting

**Figure 3:**
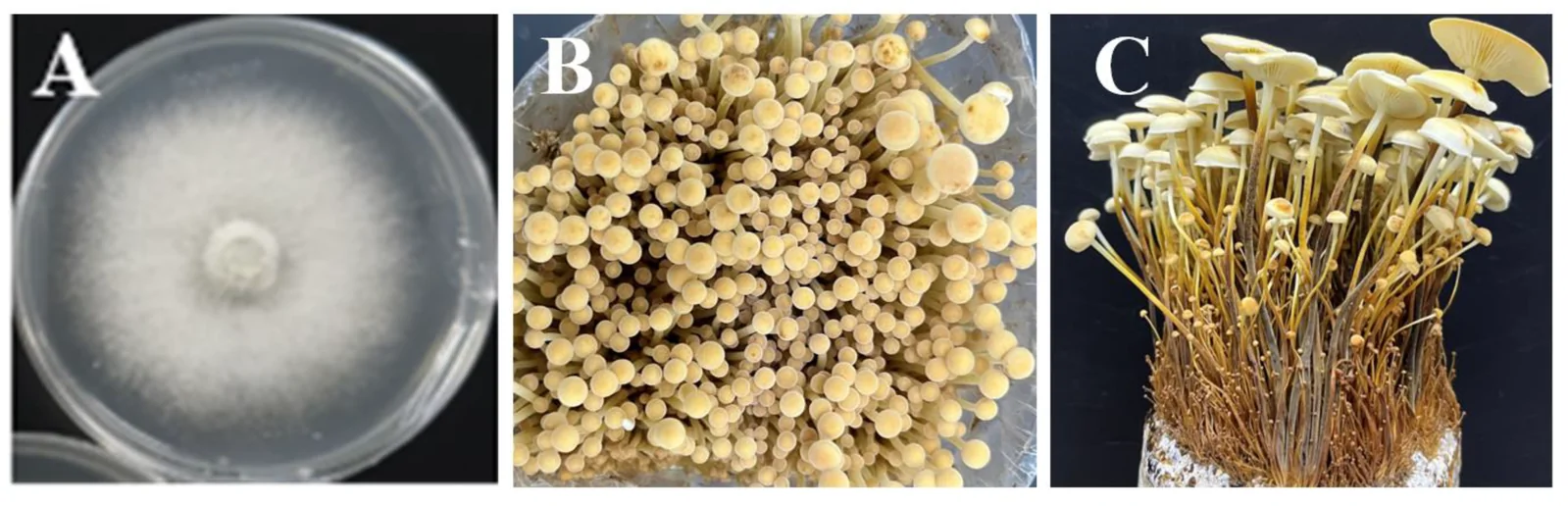
Colony and cultivated fruiting body morphology of *F. yunnanensis* strain Z68. (**A**) Mycelial colony on plate, (**B**) Primordium stage, (**C**) Mature stage.

As shown in Figure 3, strain Z68 formed white colonies on plate medium, with a flat morphology, neat margins, and dense mycelia (Figure 3A). Cultivation experiments showed that the mycelia fully colonized the cultivation bags within 20 days. Approximately 6 days after scratching, primordia appeared in clustered, concentrated, and uniform formations. After a further 10 days of cultivation, the fruiting bodies matured and were ready for harvest. The mature fruiting bodies had pale yellow caps, crisp stipes, and dark brown stipe bases (Figure 3B,C). In terms of yield, under bag cultivation with 500 g of wet substrate, the average fresh mushroom yield and dry weight were 27.81 g per bag and 3.64 g per bag, respectively. Under a substrate moisture content of 65%, the biological efficiency of strain Z68 was 15.89%.

### 3.4. Nutritional Components of Fruiting Bodies

#### 3.4.1. Nutritional Composition and Amino Acid Content of the Fruiting Body

The nutritional composition of the fruiting bodies was further analyzed to evaluate the nutritional value of strain Z68 in comparison with the control cultivars. The results in Table 6 showed that strain Z68 exhibited a significantly higher protein content (25.1 g/100 g) compared to M58 (15.5 g/100 g) and FV0027 (17.5 g/100 g), with a ~1.6- to 1.9-fold increase. This elevated protein level suggests Z68 may serve as a more valuable dietary protein source, potentially beneficial for human or animal nutrition. In contrast, fat content was the lowest in Z68 (1.1 g/100 g), while M58 (1.8 g/100 g) and FV0027 (1.4 g/100 g) had higher fat levels. Lower fat content in Z68 aligns with the trend of “leaner” nutritional profiles, which may appeal to health-conscious consumers. For dietary fiber, total dietary fiber showed minor variations among the three strains (Z68: 45.5 g/100 g; M58: 47.7 g/100 g; FV0027: 48 g/100 g), with Z68 being slightly lower. However, soluble dietary fiber in Z68 (3.8 g/100 g) was comparable to M58 (4.15 g/100 g) but higher than FV0027 (2.52 g/100 g). Soluble fiber is known to modulate gut microbiota and improve digestion, implying Z68 may have a unique advantage in gut-health promotion. The amino acid profiles of the fruiting bodies. Strain Z68 exhibited a significantly higher total amino acid content (19.80 g/100 g) compared to the control cultivars M58 (12.70 g/100 g) and FV0027 (15.20 g/100 g), representing a substantial nutritional advantage (Table 7). Among the essential amino acids, lysine and methionine were notably enriched in Z68 (1.09 g/100 g and 1.88 g/100 g, respectively), which are often limited in conventional plant-based diets and are crucial for human growth and metabolism. Furthermore, glutamate, a key contributor to the umami taste, was remarkably elevated in Z68 (3.96 g/100 g). This not only enhances the palatability of the mushroom but also indicates its potential as a flavor-enhancing ingredient in food products. Collectively, the superior amino acid profile highlights Z68 as a promising candidate for functional food development.

#### 3.4.2. Determination of Mineral and Vitamin Content

As shown in Table 8, the fruiting bodies of strain Z68 exhibited superior nutritional quality compared to the control cultivars, particularly in terms of iron, potassium, and riboflavin content. Notably, the iron content of Z68 was remarkably elevated, reaching 207 mg/kg, which was 2.9-fold and 4.1-fold higher than that of M58 and FV0027, respectively. This significant enrichment in iron suggests a potentially enhanced capability for redox regulation or a higher nutritional value for human consumption. Similarly, potassium levels in Z68 (5.30 × 10^4^ mg/kg) were approximately 1.7 times higher than the controls, contributing to electrolyte balance. In contrast, while the calcium content in Z68 (127 mg/kg) was significantly higher than FV0027 (11.4 mg/kg), it was notably lower than M58 (410 mg/kg), indicating a distinct mineral profile. Regarding vitamins, riboflavin (Vitamin B2) was the most abundant in Z68 (0.864 mg/100 g), although the differences among the three strains were less pronounced compared to minerals. Overall, these findings highlight Z68 as a promising candidate with optimized micronutrient composition, especially for iron and potassium fortification.

### 3.5. Genome Sequencing, Assembly, and Annotation of Z68

After quality filtering, Illumina paired-end sequencing yielded 68,802,704 high-quality clean reads, corresponding to approximately 10.31 Gb of total bases, with a mean read length of 149.8 bp and a Q30 base percentage of 96.17%. FastQC analysis confirmed the absence of adapter contamination and excessive unknown bases, with a GC content of 47.41% and a normal distribution profile, meeting all requirements for downstream analyses. Concurrently, PacBio sequencing generated 991,432 circular consensus sequencing (CCS) reads, totaling 14,695,773,775 bp (~14.7 Gb). The mean CCS read length was 14,823 bp, with a continuous length distribution. The CCS read N50 reached 15,700 bp, indicating that more than half of the total data were contained in reads longer than 15,700 bp. Notably, the longest single CCS read extended to 47,250 bp, reflecting the long-read capability essential for spanning repetitive genomic regions and reducing assembly fragmentation, which is particularly advantageous for de novo genome assembly and structural variant detection in high-quality genomic studies. The final genome assembly comprised 63.88 Mb in 771 contigs, with a contig N50 of 2.39 Mb and an L50 of 10; the longest contig was 4.37 Mb. The overall GC content of the assembly was 38.1%, and no gap (N) calls or quality-decayed regions were detected, indicating high base-level accuracy and assembly completeness. Genome completeness was evaluated using BUSCO against the fungi_odb10 dataset (758 single-copy orthologs). Of these, 722 complete BUSCOs were identified (completeness = 95.3%), comprising 695 single-copy (91.7%) and 27 duplicated (3.6%) genes. Only six genes were fragmented (0.8%), and 30 were missing (3.9%). These metrics demonstrate a highly complete genome assembly with reliable gene set quality, suitable for subsequent comparative genomic investigations. A total of 15,351 protein-coding genes were predicted and functionally annotated. InterPro domain assignments were obtained for 64.2% (9862) of the genes, and 57.6% (8835) showed homology to sequences in the EggNOG database, indicating that the majority possess recognizable conserved domains or evolutionary orthologs. For specific functional categorization, 32.9% (5054) of genes were assigned to KEGG Orthology (KO) identifiers, and 50.5% (7752) received Gene Ontology (GO) terms. Additionally, 453 carbohydrate-active enzymes (CAZymes, 3.0%) and 521 genes (3.4%) located within biosynthetic gene clusters (BGCs) were identified. Notably, 32.9% (5057) of the predicted genes remained annotated only as “hypothetical proteins,” suggesting a possible proportion of species-specific genes or rapidly evolving functional sequences in this organism. Overall, the integration of Illumina short-read and PacBio long-read sequencing platforms yielded a highly contiguous, gap-free genome assembly with exceptional completeness and broad functional coverage, establishing a robust and reliable reference framework for subsequent comparative genomics, evolutionary inference, and species-specific metabolic pathway discovery.

### 3.6. Iron Metabolism Gene Family Copy Numbers Across Three Flammulina Species

**Figure 4:**
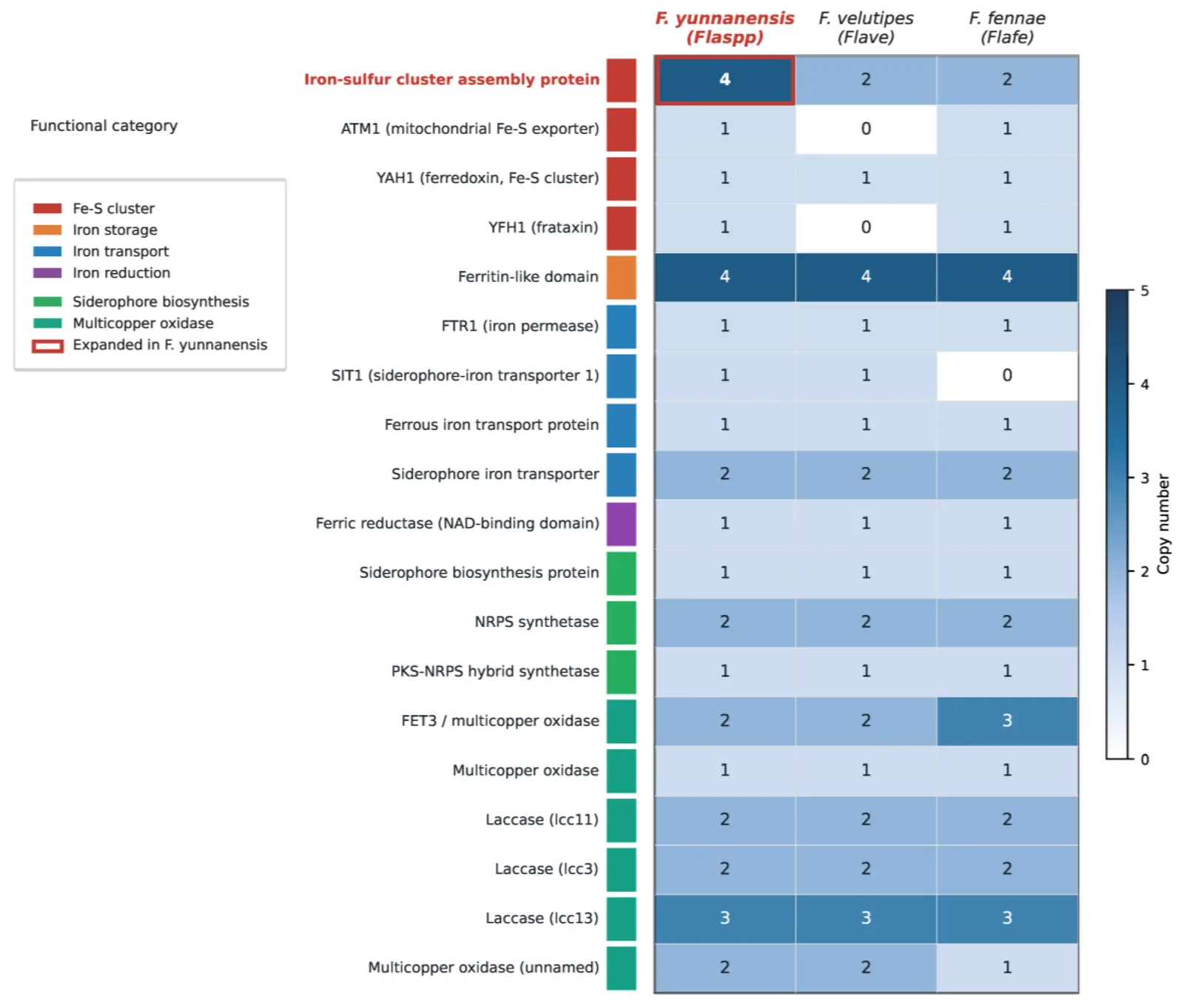
Copy numbers of 19 iron-metabolism gene families across three *Flammulina* species from OrthoFinder clustering. Note: *F. yunnanensis*/Flaspp, *F. velutipes*/Flave, *F. fennae*/Flafe. Cell shading indicates copy number (0–5). The red-outlined cell marks the Fe-S cluster assembly protein family expanded in *F. yunnanensis* (4 vs. 2 in both controls). Colour bars at left denote functional category.

OrthoFinder clustering of 19 functionally annotated iron-metabolism gene families revealed an uneven distribution across the three *Flammulina* species (Figure 4, Table 9). Most families were present as conserved single-copy orthologues in all three species, including FTR1 (iron permease, 1:1:1), the ferric reductase NAD-binding domain (1:1:1), ferredoxin YAH1 (1:1:1), the ferrous iron transport protein (1:1:1), the siderophore biosynthesis protein (1:1:1), the PKS-NRPS hybrid synthetase (1:1:1) and the multicopper oxidase (1:1:1). Several families departed from this pattern. The iron–sulfur (Fe-S) cluster assembly protein family was expanded specifically in *F. yunnanensis* (4:2:2; a 2-fold expansion relative to both controls; Figure 4, red-outlined cell), whereas FET3/multicopper oxidase was expanded in *F. fennae* (3 vs. 2) and the unnamed multicopper oxidase family was reduced in *F. fennae* (1 vs. 2). Three families showed lineage-specific loss: ATM1 (mitochondrial Fe-S exporter, 1:0:1) and YFH1 (frataxin, 1:0:1) were both absent in *F. velutipes*, and SIT1 (siderophore-iron transporter 1, 1:1:0) was absent in *F. fennae*. The Fe-S cluster assembly family encompasses scaffold proteins (e.g., IscU/SufU) and assembly factors required for biogenesis of [2Fe-2S] and [4Fe-4S] clusters [55,56], which serve as cofactors for mitochondrial respiratory complexes and cytosolic iron sensors; its expansion in *F. yunnanensis* is consistent with an enhanced Fe-S biogenesis capacity and may reflect higher intracellular iron demand. The concurrent loss of ATM1 and YFH1 in *F. velutipes* is consistent with reduced Fe-S export and storage capacity in that species. Critically, despite the ~3–4-fold higher measurable iron content of *F. yunnanensis*, FTR1 was single-copy in all three species (1:1:1), indicating that the iron content phenotype is not explained by gene dosage at this locus. This observation redirected attention to functional differences at the protein sequence level, explored in the subsequent analyses.

### 3.7. Species-Specific Amino Acid Substitutions in FTR1 Iron Permease

MAFFT alignment identified 13 variable positions in FTR1, of which 6 involved a physicochemical property change unique to *F. yunnanensis* (Table 10). All three ColabFold/AlphaFold2 models showed comparable mean pLDDT scores (76.9–77.4), with the seven transmembrane helices consistently exhibiting the highest per-residue confidence (predominantly >80), indicating well-converged, structurally comparable 7-TM folds across the three species. The six property-changing substitutions therefore represent localised functional modifications within an otherwise conserved scaffold.

#### 3.7.1. Position 372: Glu→Lys: Charge Reversal at the Intracellular Exit (KEY)

*F. yunnanensis* carries Lys (+1) at the C-terminal intracellular tail (DeepTMHMM: inside, residues 337–382), replacing Glu (−1) in both controls (Δcharge = +2; pLDDT = 69.6). Based on the established FTR1 mechanism in which Fe^3+^ is translocated through the channel and released as Fe^2+^ into the cytoplasm [57,58], the positive charge at the exit site is predicted to repel Fe^2+^ and accelerate its release, whereas the Glu in control species would attract and retain Fe^2+^, slowing turnover. As this conclusion rests on sequence evidence, it holds independently of the moderate structural confidence at this disordered region.

#### 3.7.2. Position 127: Ser→Ala: Pore Widening in TM3 (Highest Confidence)

Within TM3 (DeepTMHMM: residues 120–137), *F. yunnanensis* carries Ala (nonpolar, ≈ 67 Å^3^) where both controls carry Ser (polar hydroxyl, ≈77 Å^3^; pLDDT = 91.6, the highest among all variable sites). Removing the serine hydroxyl reduces the side-chain volume by ~10 Å^3^ and eliminates a potential hydrogen-bond donor at this pore-facing position. On this basis, the Ser→Ala substitution is tentatively hypothesised to modestly widen the TM3 lumen and lower the steric/desolvation barrier to iron permeation. This inference rests on sequence and side-chain-volume comparison only and has not been tested; direct support would require site-directed mutagenesis in an ftr1 background.

#### 3.7.3. Positions 358 and 365: Remodeling of the C-Terminal Intracellular Vestibule

Ile-358 (nonpolar) replaces polar Thr/Asn in controls (pLDDT = 78.8), reducing local hydrogen-bond density near the TM7 exit. Gln-365 (polar neutral) replaces His (pK_a_ ≈ 6) in controls (pLDDT = 81.6); histidine is a canonical metal-coordination residue [59] whose replacement eliminates both partial positive charge and Fe^2+^-binding capacity at the exit vestibule. These two substitutions act synergistically with Lys-372 to simplify the intracellular exit environment and facilitate rapid iron release.

#### 3.7.4. Positions 40 and 378: Tentative Implications (Low Structural Confidence)

Lys-40 (pLDDT = 55.3; extracellular loop) introduces a positive charge that may facilitate electrostatic capture of negatively charged siderophore-Fe complexes at the channel entry. Ile-378 (pLDDT = 50.5) replaces Pro in *F. velutipes*, potentially relieving backbone rigidity at the C-terminal tail. Both sites are intrinsically disordered, and these inferences require experimental validation.

## 4. Discussion

The growth of edible mushroom mycelia is influenced by multiple factors, including temperature, nutrient availability, and pH. Appropriate temperature, suitable carbon and nitrogen sources, and an adequate supply of inorganic salts are all essential for rapid mycelial growth [60,61]. Therefore, investigating the basic biological characteristics of a strain is a prerequisite for the artificial domestication and efficient cultivation of wild edible mushroom resources [62].

In the present study, single-factor experiments were conducted to systematically evaluate the effects of carbon source, nitrogen source, inorganic salt, pH, and temperature on the mycelial growth of *F. yunnanensis* strain Z68. The results showed that maltose was the most suitable carbon source for Z68. This finding is not entirely consistent with previous reports on *Flammulina* species. For example, Feng et al. [62] reported that fructose was the optimal carbon source for *F. velutipes* JC42, whereas Lu Xinxin et al. [63] found that glucose was the most favorable carbon source for *F. velutipes* strain 21JYF01. He Jun et al. [20] suggested that malt extract powder was the most suitable carbon source for Liusheng *F. velutipes*, while Wang Xin et al. [64] identified soluble starch as the optimal carbon source for *F. yunnanensis*. These discrepancies indicate that carbon source preference and utilization efficiency may differ not only among species, but also among strains within the same genus. Therefore, culture medium formulations should be optimized according to the biological characteristics of specific strains in practical cultivation. It is also noteworthy that Z68 still exhibited a certain degree of growth on the carbon-free control medium. This may be attributable to trace utilizable carbon present in the agar or to the consumption of nutrients stored within the mycelia themselves. However, the sparse mycelial growth observed under this condition further indicates that high-quality exogenous carbon sources remain essential for vigorous mycelial development.

The nitrogen source experiment demonstrated that yeast extract was the optimal nitrogen source for strain Z68, whereas urea was poorly utilized. This result is generally consistent with previous studies on *Flammulina* mushrooms and suggests that species of this genus tend to preferentially utilize readily available organic nitrogen sources [65,66].

The pH experiment showed that the optimal pH range for mycelial growth of Z68 was 7.0–8.0, which differs somewhat from previous reports indicating that the optimal pH for *Flammulina* mycelia is usually within a slightly acidic to neutral range [67,68,69]. This result suggests that Z68 is well adapted to neutral to weakly alkaline conditions, and such a difference may be associated with its genetic background and ecological origin. In addition, the temperature experiment revealed that the optimal growth temperature for Z68 was 20 °C. Previous studies have shown that the optimal temperature for mycelial growth in *Flammulina* is generally within the range of 20–25 °C, and that many cultivated strains grow well at 23–25 °C [11,70,71]. Since Z68 was isolated from the Tibetan Plateau, its relatively low optimal growth temperature may reflect long-term adaptation to the cool climatic conditions of its native habitat. This finding implies that, during the domestication of wild strains, the ecological characteristics of the original habitat should be fully considered so that cultivation conditions can be adjusted accordingly.

In terms of fruiting cultivation, strain Z68 exhibited relatively favorable cultivation performance [26]. Nutritional analysis further showed that the protein content of its fruiting bodies reached 25.1 g/100 g, while the total content of 16 amino acids reached 19.80 g/100 g, both of which were significantly higher than those of the two control cultivars. These results indicate that Z68 possesses a strong capacity for nutrient accumulation. Lei et al. [26], in a comparative study of five *Flammulina* species, reported that *F. yunnanensis* GS0107 had a relatively high crude protein content within the genus and that nutritional traits varied markedly among species. This is broadly consistent with the high-protein and high-amino-acid characteristics observed in Z68 in the present study [72]. Regarding amino acid composition, previous studies have shown that the major amino acids in *Flammulina* fruiting bodies are generally aspartic acid, glutamic acid, and lysine, among which glutamic acid and aspartic acid are closely related to umami taste [64]. In Z68, glutamic acid was the most abundant amino acid and was present at a level significantly higher than that reported for Jzg-1, suggesting that Z68 may possess desirable flavor quality [73]. Nevertheless, the amino acid profile of Z68 still differs somewhat from those reported for other *F. yunnanensis* materials, implying that amino acid metabolism may vary among strains of the same species.

Mineral element analysis further demonstrated that Z68 has clear advantages in mineral nutrient accumulation. Previous studies have shown that *Flammulina* species generally exhibit a low-sodium, high-potassium nutritional profile, while the contents of trace elements such as iron and zinc vary substantially among species. Consistent with this pattern, potassium was the most abundant mineral element in Z68 fruiting bodies, reaching 5.30 × 10^4^ mg/kg, which was 15 times that reported for GS0107 [26] and also significantly higher than those of the two control cultivars. This result indicates that Z68 retains the characteristic high-potassium enrichment commonly observed in *Flammulina*. At the same time, the zinc and iron contents in Z68 were also markedly higher than those in the control strains, highlighting its potential advantages in trace element accumulation.

FTR1 encodes a high-affinity iron permease that plays a central role in iron uptake and cellular homeostasis, where a link between FTR1, iron availability, and physiological responses exists. In this research, two-fold expanded iron permease FTR1 in *F. yunnanensi* was discovered, which is the iron–sulfur cluster assembly protein family. Sequence, topology (DeepTMHMM), and structural-confidence (ColabFold/AlphaFold2) analyses of FTR1 identified six species-specified physicochemical substitutions, of which a charge reverse at the intracellular channel (Glu→Lys) and a polar-to-nonpolar change in the TM3 pore (Ser→Ala) were predicted to enhance iron transport efficiency. These suggested a possible two-tier mechanism underlying the elevated iron content of *F. yunnanensis*, combining gene-family expansion and predicted functional fine-tuning of the FTR1 permease. In addition, FTR1-mediated iron uptake may be indirectly linked to oxidative-stress responses. Because Ftr1 functions as a component of the high-affinity iron uptake system [74], increased iron influx may enlarge the intracellular labile iron pool, thereby promoting Fenton-type reactions and reactive oxygen species production. Consistent with this possibility, high intracellular iron levels have been shown to induce ROS accumulation and activate antioxidant defense systems, such as alternative oxidases and, as reported in related studies, catalase and superoxide dismutases—in fungi [75].

## 5. Conclusions

In summary, the fruiting bodies of strain Z68 contained significantly higher levels of protein, multiple essential amino acids, and mineral elements such as potassium, zinc, and iron than those of the control strains *F. fennae* M58 and *F. velutipes* FV0027, demonstrating clear nutritional advantages. In particular, its high protein content, high potassium level, and relatively rich essential amino acid composition indicate that Z68 has considerable nutritional value. Moreover, its mineral profile, characterized by high potassium and low sodium, suggests potential application in salt-restricted diets, which may contribute to electrolyte balance and help reduce the risk of hypertension and cardiovascular diseases [76]. In addition, whereas previous studies on *Flammulina* have mainly focused on protein, fat, crude fiber, amino acids, and mineral elements, the present study further determined the contents of vitamin B1, vitamin B2, and L(+)-ascorbic acid, thereby broadening the scope of nutritional quality evaluation for *F. yunnanensis* to some extent. To gain initial insight into the molecular basis of the notably higher iron content observed in Z68, we performed comparative genomic and structural bioinformatics analyses among three *Flammulina* species. These analyses ruled out FTR1 gene dosage effects (single-copy in all species) but identified a lineage-specific expansion of the Fe-S cluster assembly protein family (4 copies in *F. yunnanensis* vs. 2 copies in controls) and six species-specific amino acid substitutions in the FTR1 iron permease, including a Ser→Ala change in the TM3 pore region (tentatively suggesting a lower permeation barrier) and a Glu→Lys charge reversal in the C-terminal intracellular tail (predicted to accelerate Fe^2+^ release via electrostatic repulsion). It must be emphasised that these functional interpretations are derived solely from sequence alignments, topology predictions and AlphaFold2 models; they remain hypotheses that await experimental validation (e.g., functional complementation in an ftr1Δ yeast background and directed iron transport assays). Overall, the present study provides a preliminary basis for the conservation, domestication, and further utilization of wild *F. yunnanensis* germplasm resources.

## Figures and Tables

**Table 1:** Effect of Different Carbon Sources on the Mycelial Growth of *F. yunnanensis* Strain Z68.

Cultivation Condition	Factors	Speed of Growth (mm/d) *	Hyphal Density **
Carbon source	CK (basal medium)	6.98 ± 0.16 ^bA^	+
	Fructose	7.27 ± 0.31 ^abA^	++++
	Lactose	4.11 ± 0.25 ^cB^	+++
	Maltose	7.52 ± 0.50 ^aA^	+++
	Glucose	6.98 ± 0.13 ^bA^	++++
	Sucrose	7.16 ± 0.21 ^abA^	+++

* Statistical significance was determined using Duncan’s new multiple range test; lowercase letters following data in the same column indicate differences at the *p* < 0.05 level, and uppercase letters indicate differences at the *p* < 0.01 level; values sharing the same letter do not differ significantly. ** hyphal densities were scored (thin, +; dense, +++; compact, ++++).

**Table 2:** Effect of Different Nitrogen Sources on the Mycelial Growth of *F. yunnanensis* Strain Z68.

Cultivation Condition	Factors	Speed of Growth (mm/d) *	Hyphal Density **
Nitrogen source	CK (basal medium)	4.14 ± 0.17 ^dD^	+
	urea	3.75 ± 0.47 ^dD^	+
	tryptone	6.93 ± 0.25 ^bB^	+++
	Acid-hydrolyzed casein	6.45 ± 0.33 ^bcBC^	++
	Beef extract	5.99 ± 0.46 ^cC^	++
	Yeast powder	8.82 ± 0.20 ^aA^	++++

* Statistical significance was determined using Duncan’s new multiple range test; lowercase letters following data in the same column indicate differences at the *p* < 0.05 level, and uppercase letters indicate differences at the *p* < 0.01 level; values sharing the same letter do not differ significantly. ** hyphal densities were scored (thin, +; moderate, ++; dense, +++; compact, ++++).

**Table 3:** Effect of Different Inorganic Salts on the Mycelial Growth of *F. yunnanensis* Strain Z68.

Cultivation Condition	Factors	Speed of Growth (mm/d) *	Hyphal Density **
Inorganic salt	CK (basal medium)	7.51 ± 0.51 ^bcAB^	+++
	Potassium dihydrogen phosphate	7.21 ± 0.27 ^cB^	+++
	Magnesium sulfate	8.23 ± 0.50 ^abAB^	++++
	Ferrous sulfate	0.74 ± 0.94 ^dC^	−
	Calcium chloride	8.34 ± 0.22 ^aA^	+++
	Calcium carbonate	7.53 ± 0.23 ^bcAB^	+++

* Statistical significance was determined using Duncan’s new multiple range test; lowercase letters following data in the same column indicate differences at the *p* < 0.05 level, and uppercase letters indicate differences at the *p* < 0.01 level; values sharing the same letter do not differ significantly. ** hyphal densities were scored (no-growth, −; dense, +++; compact, ++++).

**Table 4:** Effect of Different pH Values on the Mycelial Growth of *F. yunnanensis* Strain Z68.

Cultivation Condition	Factors	Speed of Growth (mm/d) *	Hyphal Density **
pH	CK (basal medium)	6.91 ± 0.29 ^cD^	++++
	4.5	4.52 ± 0.23 ^eF^	++
	5.0	6.28 ± 0.13 ^dE^	++
	6.0	7.07 ± 0.31 ^cCD^	++
	7.0	7.61 ± 0.07 ^bAB^	++++
	8.0	7.74 ± 0.10 ^abAB^	+++
	9.0	7.48 ± 0.28 ^bBC^	++
	10.0	8.03 ± 0.24 ^aA^	++

* Statistical significance was determined using Duncan’s new multiple range test; lowercase letters following data in the same column indicate differences at the *p* < 0.05 level, and uppercase letters indicate differences at the *p* < 0.01 level; values sharing the same letter do not differ significantly. ** hyphal densities were scored (moderate, ++; dense, +++; compact, ++++).

**Table 5:** Effect of Different Temperatures on the Mycelial Growth of *F. yunnanensis* Strain Z68.

Cultivation Condition	Factors	Speed of Growth (mm/d) *	Hyphal Density **
Temperature/°C	12	3.67 ± 0.33 ^eE^	++
	16	4.72 ± 0.14 ^dD^	+++
	18	5.44 ± 0.31 ^cC^	+++
	20	6.79 ± 0.12 ^aA^	++++
	22	6.15 ± 0.28 ^bB^	+++
	25	2.75 ± 0.09 ^fF^	+
	28	0 ^gG^	−

* Statistical significance was determined using Duncan’s new multiple range test; lowercase letters following data in the same column indicate differences at the *p* < 0.05 level, and uppercase letters indicate differences at the *p* < 0.01 level; values sharing the same letter do not differ significantly. ** hyphael densities were scored (no-growth, −; thin, +; moderate, ++; dense, +++; compact, ++++).

**Table 6:** Comparison of basic nutritional components in fruiting bodies of *F. yunnanensis* Z68 and control cultivars.

Component/g/100 g	Z68	M58	FV0027
Protein	25.1	15.5	17.5
Fat	1.1	1.8	1.4
Total Dietary Fiber	45.5	47.7	48
Soluble Dietary Fiber	3.8	4.15	2.52

**Table 7:** Comparison of amino acid contents in fruiting bodies of *F. yunnanensis* Z68 and control cultivars.

Amino Acid/g/100 g	Strains		
	Z68	M58	FV0027
Alanine	1.28	0.78	1.09
Serine	1.02	0.65	0.80
Leucine	1.28	0.86	1.04
Glycine	0.93	0.60	0.73
Arginine	1.12	0.60	0.77
Histidine	0.46	0.25	0.34
Valine	1.12	0.68	0.84
Proline	0.52	0.40	0.48
Threonine	1.03	0.65	0.81
Methionine	1.88	1.70	1.54
Glutamate	3.96	2.13	2.76
Lysine	1.09	0.72	0.93
Tyrosine	0.68	0.47	0.55
Aspartate	1.70	1.04	1.20
Isoleucine	0.86	0.58	0.70
Phenylalanine	0.85	0.56	0.66
Total	19.80	12.70	15.20

**Table 8:** Comparison of mineral elements and vitamin contents in fruiting bodies of *F. yunnanensis* Z68 and control cultivars.

Composition		Strains		
		Z68	M58	FV0027
Mineral elements/mg/kg	Potassium	5.30 × 10^4^	3.07 × 10^4^	3.22 × 10^4^
	Calcium	127	410	11.4
	Magnesium	1.95 × 10^3^	1.35 × 10^3^	1.58 × 10^3^
	Zinc	45.4	22.3	28.1
	Iron	207	70.7	50.5
Vitamins/mg/100 g	Thiamine	0.078	0.168	0.233
	Riboflavin	0.864	0.73	1.09
	Ascorbic acid	122	130	135

**Table 9:** Copy numbers of iron metabolism gene families across three *Flammulina* species.

Gene Family	Flaspp	Flave	Flafe	Pattern	Functional Category
Iron–sulfur cluster assembly protein	4	2	2	Expanded in Flaspp	Fe-S cluster assembly
ATM1 (mitochondrial Fe-S exporter)	1	0	1	Absent in Flave	Fe-S cluster export
YAH1 (ferredoxin)	1	1	1	Conserved	Fe-S cluster
YFH1 (frataxin)	1	0	1	Absent in Flave	Iron storage/Fe-S
Ferritin-like domain	4	4	4	Conserved	Iron storage
FTR1 (iron permease)	1	1	1	Conserved (functional variants)	Iron transport
SIT1 (siderophore-iron transporter 1)	1	1	0	Absent in Flafe	Iron transport
Ferrous iron transport protein	1	1	1	Conserved	Iron transport
Siderophore iron transporter	2	2	2	Conserved	Iron transport
Ferric reductase (NAD-binding)	1	1	1	Conserved	Iron reduction
Siderophore biosynthesis protein	1	1	1	Conserved	Siderophore biosynthesis
NRPS synthetase	2	2	2	Conserved	Siderophore biosynthesis
PKS-NRPS hybrid synthetase	1	1	1	Conserved	Siderophore biosynthesis
FET3/multicopper oxidase	2	2	3	Expanded in Flafe	Multicopper oxidase
Multicopper oxidase	1	1	1	Conserved	Multicopper oxidase
Laccase (lcc11/lcc3/lcc13)	2/2/3	2/2/3	2/2/3	Conserved	Multicopper oxidase
Multicopper oxidase (unnamed)	2	2	1	Slightly reduced in Flafe	Multicopper oxidase

**Table 10:** *F. yunnanensis*-specific amino acid substitutions in FTR1 iron permease. Note: Position numbering based on MAFFT alignment; for *F. yunnanensis* raw sequence positions ≥41, alignment coordinates are offset by −1. pLDDT scores from ColabFold/AlphaFold2 (extracted from PDB B-factor column). Topology predicted by DeepTMHMM. Labels in Topology column: [High-confidence]—structurally critical region (TM3 core) with high pLDDT; [Property-changing]—substitution alters charge, polarity, or metal coordination; [Conservative]—functionally neutral; [tentative]—low pLDDT (<70) or disordered region. Symbols: ★★★ highest confidence, ★★ moderate, ★ suggestive. KEY indicates predicted major functional impact (charge reversal in C-terminal tail).

Pos.	Flaspp	Flave	Flafe	pLDDT	Physicochemical Change	Topology/Significance
372	K	E	E	69.6	Acidic→Basic (Δcharge = +2)	[Property-changing]C-terminal intracellular tail ★★ KEY
127	A	S	S	91.6	Polar→Nonpolar (hydroxyl removed)	[High-confidence]TM3 core ★★★
358	I	T	N	78.8	Polar→Nonpolar	[Property-changing]C-terminal intracellular tail ★
365	Q	H	H	81.6	Basic→Polar; loss of His metal-coordination	[Property-changing]C-terminal intracellular tail ★
40	K	T	T	55.3	Polar→Basic (+charge); disordered	[Property-changing tentative]N-terminal extracellular loop ★
378	I	P	L	50.5	Pro (rigid)→Ile; disordered	[Property-changing tentative]C-terminal intracellular tail ★
45–53, 222, 366	(various)	(various)	(various)	78–84	Conservative (same property class)	[Conservative]Various loops—no functional impact

## Data Availability

No new data were created or analyzed in this study. Data sharing is not applicable to this article.
